# Effect of Different Salts on Nutrients Uptake, Gene Expression, Antioxidant, and Growth Pattern of Selected Rice Genotypes

**DOI:** 10.3389/fpls.2022.895282

**Published:** 2022-06-16

**Authors:** Muhammad Farooq, Saleem Asif, Yoon-Hee Jang, Jae-Ryoung Park, Dan-Dan Zhao, Eun-Gyeong Kim, Kyung-Min Kim

**Affiliations:** ^1^Department of Applied Biosciences, Graduate School, Kyungpook National University, Daegu, South Korea; ^2^Crop Breeding Division, National Institute of Crop Science, Rural Development Administration, Wanju, South Korea

**Keywords:** *OsHKT1*, *OsNHX1*, *OsSOS1*, macronutrients, micronutrients, CaCl_2_, MgCl_2_

## Abstract

Climate change leads to soil salinization, and the dynamic scarcity of freshwater has negatively affected crop production worldwide, especially *Oryza sativa*. The association among ion uptake, gene expression, antioxidant, biomass, and root and shoot development under different salt stress are not fully understood. Many studies are related to the effect of NaCl only. This study used two salts (CaCl_2_ and MgCl_2_) along with NaCl and analyzed their effects on mineral uptake (macronutrients and micronutrients), gene expression, seed germination, antioxidants, plant growth, and biomass in different rice genotypes. CaCl_2_ (up to 200 mM) slightly increased the germination percentage and seedling growth, whereas, 150 mM MgCl_2_ in the soil increased the root, shoot length, and fresh and dry weight in cultivars IR 28 and Cheongcheong. All agronomic traits among rice genotypes were drastically reduced by NaCl stress compared to other salts. Different salt stress differentially regulated ion uptake in the roots and shoots among different rice genotypes. Under different salt stress, a consistent decrease in Ca^2+^, Mn^2+^, and Fe^2+^ ions was observed in the roots of Cheongcheong, Nagdong, and IR 28. Similarly, under different salts, the stress in the shoots of Cheongcheong (Ca^2+^, Na^+^, and Zn^2+^) and Nagdong (Ca^2+^, Mg^2+^, Na^+^, and Zn^2+^) and the shoots of IR 28 (Ca^2+^ and Mg^2+^) consistently increased. Under different salts, a salt stress-related gene was expressed differentially in the roots of rice genotypes. However, after 6 and 12 h, there was consistent *OsHKT1*, *OsNHX1*, and *OsSOS1* gene upregulation in the shoots of Nagdong and roots and shoots of the salt-tolerant cultivar Pokkali. Under different salt stress, glutathione (GSH) content increased in the shoot of IR 28 and Nagdong by NaCl, and MgCl_2_ salt, whereas, POD activity increased significantly by CaCl_2_ and MgCl_2_ in cultivar Cheongcheong and IR 28 shoot. Therefore, this study suggested that Pokkali responded well to NaCl stress only, whereas, the plant molecular breeding lab cultivar Nagdong showed more salt tolerance to different salts (NaCl, CaCl_2_, and MgCl_2_). This can potentially be used by agriculturists to develop the new salt-tolerant cultivar “Nagdong”-like Pokkali.

## Introduction

Rice (*Oryza sativa* L.) is the main cereal crop worldwide with respect to cultivated area and total production. It provides almost 20% of the world’s dietary energy supply. Salinity is a crucial abiotic ecological factor that decreases rice and other plant growth and productivity worldwide. Soil salinity affects agriculture productivity in several regions globally, including Australia, Argentina, China, Egypt, Iran, Iraq, Pakistan, Thailand, and the United States [i.e., an area of >800 million ha; Rengasamy (2010)]. An estimated 5% or 3.85 million hectares of the total cultivated area in the world is affected by salinity (Sheng et al., 2008). By 2050, salt-affected soil is predicted to be increased up to 16.2 million ha, resulting in food insecurity for the world’s population (Yadav et al., 2017).

Soil is considered saline when it exhibits an apparent electrical conductivity of >4 ds m^–1^ (Daliakopoulos et al., 2016). Naturally, soil contains various kinds of soluble salts. Increasing salt concentration imposes ionic and osmotic stress on plants, causing several morphological and physiological changes. High salt content, particularly Cl^–^ and Na^+^ sulfates, alters plant growth by modifying their anatomical, morphological, and physiological traits (Muscolo et al., 2003). Many studies reported that salt stress leads to a decrease in xylem development (Hilal et al., 1998). Some studies suggested that salt stress negatively decreases the root and shoot system’s fresh and dry weight by changing their concentration (Taffouo et al., 2010). The major cations found from soluble salts in saline soils consist of Na^+^, Ca^2+^, and Mg^2+^, whereas the most common anions are chloride, sulfates, and carbonates (including bicarbonates). Among various salts, sodium chloride (NaCl) is the most familiar worldwide. Most research studying plant physiological responses to salinity depends on experiments on NaCl stress only (Chinnusamy et al., 2005).

Salt stress activates the expression of various osmoresponsive genes and proteins in rice tissue (Chourey et al., 2003). The response of rice to salinity varies with their growth phases. Therefore, most regularly cultivated rice cultivar seedlings are very sensitive to salinity (Lutts et al., 1995; Zeng and Shannon, 2000). Thus, it can cause reductions in final germination percentage, germination energy percentage, and germination speed and lead to reduced root/shoot growth and dry matter (Ologundudu et al., 2014). Rice is considered the most sensitive crop for salinity stress at various growth stages. Salinity stress significantly damages the root surface area. Normal root growth needs essential elements, including Ca^2+^, Mg^2+^, Fe^2+^, and Zn^2+^, which are affected during salt stress. Thus, the reduction in root growth might influence Ca^2+^, Mg^2+^, Fe^2+^, and Zn^2+^ uptake (Robin et al., 2016). Quinoa seeds demonstrated decreased Ca^2+^, Mg^2+^, Zn^2+^, and Mn^2+^ contents in response to saline-sodic soil [e.g., in Larissa, Greece croplands; Karyotis et al. (2003)]. Some studies reported that salinity stress decreases Mn levels in the shoots of corn (Rahman et al., 1993). In contrast, some studies suggested that salt stress had no effect (Al-Harbi, 1995) or increased Mn content in the shoot or leaf tissue (Niazi and Ahmed, 1984). Most studies reported that salinity stress enhances Zn^2+^ concentrations in the shoots of maize (Rahman et al., 1993), citrus (Ruiz et al., 1997), and tomato (Knight et al., 1992). However, it was decreased in cucumber leaves (Al-Harbi, 1995). Some studies showed that salinity stress has an antagonistic effect on Ca^2+^, K^2+^, Fe^2+^, Mn^2+^, P, and Zn^2+^ but has a synergistic effect on Mg and N in rice (Jung et al., 2009; García et al., 2010).

After the salt overly sensitive (SOS) pathway proteins, another high-affinity K^+^ transporter (HKT) family of monovalent ion transporters is known as a relevant component in the plant defense against increased salinity levels (Rus et al., 2001, 2006). However, the main function of plant ion transporters in the HKT family remains unspecific. HKT1 was first confined and described in wheat (*Triticum aestivum*) as an HKT with Na^+^/K^+^ content carrier (Rubio et al., 1995). However, in *Arabidopsis thaliana*, the HKT1 functions as selective Na^+^ transporter (Uozumi et al., 2000). Salinity stress-induced cellular Ca^2+^ is considered a starting point in the SOS pathway for Na^+^ efflux facilitation. Furthermore, it forms a complex between a calcium-binding protein, SOS3 (CBL4), and a protein kinase, SOS2. Phosphorylation can occur between the SOS3/SOS2 complex and thus activate the Na^+^/H^+^ antiporter SOS1, which enhances Na^+^ export from cells. The Na^+^ export is not restricted only to the root surface, but their redistribution is more important to the whole body of the plant (Shi et al., 2000, 2003; Qiu et al., 2002; Oh et al., 2009). The Na^+^/H^+^ antiporter *AtNHX* family is present in *A. thaliana*, which plays a positive role in Na^+^ compartmentation (Blumwald, 2000). The Na^+^/H^+^ antiporter genes *OsNHX1* to *OsNHX5* have also been identified in rice (*O. sativa* L.; Fukuda et al., 2011). Across cell membranes, these genes play a pivotal role in catalysis to exchange Na^+^ for H^+^ that regulates cell volume, internal pH, and Na^+^ levels in the cytoplasm. Antiporter genes are also found in bacteria, yeast, animals, and plants with localization mainly in the cell membrane (Orlowski and Grinstein, 1997) and other organelles along with the perivacuolar compartment (Nass and Rao, 1998).

Salt stress, increases the formation of ROS within plant cells, and its high accumulation leads in oxidative damage of membrane lipids, protein, and nucleic acids (Ali et al., 2017; Sahin et al., 2018). There are some Enzymatic and non-enzymatic antioxidant which play a crucial role to minimize high ROS levels, an efficient plant system (Karuppanapandian et al., 2011; Chawla et al., 2013). Glutathione (GSH; c-glutamyl-cysteinyl-glycine) is a small intracellular thiol molecule which is considered as a strong non-enzymatic whereas peroxidase (POD) is enzymatic antioxidant (Foyer and Noctor, 2011; Hasanuzzaman et al., 2017). Higher GSH levels retain osmotic balance by induction of osmoprotectants and they prevent damage to, amino acids, lipids, and polysaccharides, thereby preventing damage to membranes, mitochondria, photosynthetic pigments, and other organelles which ultimately show tolerance to abiotic stresses (Nahar et al., 2015a,b,c). PODs function not only concern in scavenging H_2_O_2_ but also in plant growth, development, lignification, suberization, and cross-linking of cell wall compounds (Passardi et al., 2005). Higher activity of POD and some other antioxidants has been observed in salt tolerant cultivars of Calendula, Jatropha, pea, and tomato (Hernandez et al., 2000; Chaparzadeh et al., 2004; Mittova et al., 2004; Gao et al., 2008).

The *O. sativa* cultivar Nipponbare genome consists of nine *HKT*, five *OsNHX1*, and three genes of the SOS1 family. These genes play a crucial role in ion balance during salinity stress. Therefore, one gene from each family was selected in this study, and their relative gene expression was checked at different time points under different salt stress. However, different species varieties or cultivars have different salinity tolerance mechanisms. This study tested the two-parent cultivars, Cheongcheong and Nagdong of Cheongcheong/Nagdong Doubled haploid population (CNDH), with salt-sensitive cultivar IR 28 and salt-tolerant cultivar Pokkali. Most studies focused only on NaCl stress to check the plant physiological response against NaCl. However, this study investigated the effect of different salts, such as NaCl, CaCl_2_, and MgCl_2_, on the uptake of macronutrients and micronutrients, growth index, and gene expression in relation to salinity in four different rice cultivars at the early vegetative stage. The findings could help determine which salt most seriously affected growth attributes, ion uptake, and gene expression among different rice cultivars. Furthermore, the findings could help develop new salt-tolerant or salt-sensitive cultivars in the future for plant molecular breeding in the laboratory using genome editing techniques.

## Materials and Methods

### Plant Material and Preparation of Brine Solution

Seeds of four different rice cultivars, Cheongcheong, Nagdong, IR 28, and Pokkali, were used in the experiment. IR 28 is a salt-sensitive cultivar, and Pokkali is a salt-tolerant cultivar used worldwide, whereas cultivars Cheongcheong and Nagdong are two famous cultivars of plant molecular breeding laboratories in South Korea. Three different salts NaCl, CaCl_2_, and MgCl_2_ with concentrations of 100, 150, 200, and 250 mM were used for seed germination and seedling analysis of four different rice genotypes.

### Seed Germination and Seedling Analysis

Before germination, seeds of four rice genotypes were surface sterilized with 70% ethanol for 1 min, followed by 5% sodium hypochlorite for 20 min. Afterward, seeds were washed five times with double-distilled water and dried for 40 min using autoclaved filter paper inside a clean bench. Small Petri dishes (∼9 cm in diameter) containing autoclaved filter paper were used for germination tests; 45 seeds were placed in a single Petri dish to give three replicates for each cultivar and treatment. Different salt treatments (NaCl, CaCl_2_, and MgCl_2_) were made at concentrations of 100, 150, 200, and 250 mM. The seeds were kept for a 14 h light/10 h dark photoperiod at 28°C in the light and dark. Relative humidity was maintained at 60% inside the growth room. The different salt stress treatments lasted for 2 weeks. Seeds were considered to have germinated when the radical was protruding from the seed coat. Germinated seeds were counted after each week. Finally, after 14 days, germination rate and seedling growth were recorded phenotypically.

### Seedling Analysis in Soil Using Different Salt Treatments

Four rice genotypes were pregerminated and grown in small water cups of 500 mL (Σ100 mm in diameter and 120 mm in height) containing soil for 4 weeks in the greenhouse. Before salinity stress, the pH and EC of the soil were measured (4.5–5.5 and 2.0, respectively). Each water cup contains 10–12 germinated seedlings and makes three replicates of each salt treatment for four rice cultivars. Therefore, when rice seedlings reach the four- or five-leaf stage, 150-mM NaCl, CaCl_2_, and MgCl_2_ salts (200 mL) were applied twice daily for 7 days. Afterward, phenotypic differences were observed among rice cultivars, and the root and shoot length of the rice seedlings were measured.

### Measurement of Fresh and Dry Weights

After salinity stress with 150-mM NaCl, CaCl_2_, and MgCl_2_, roots, and shoots from rice cultivars were collected, and the fresh weight was measured. Furthermore, the samples were dried at 65°C for 5 days, and the root and shoot dry weight was determined.

### Ion Determination

Roots and shoots of four rice cultivars were used for ion analysis, as described by Rus et al. (2001) with slight modification. Briefly, the samples were dried at 65°C for 7 days and ground immediately in liquid nitrogen. Furthermore, 100-mg powder was extracted with 10 mL of 0.1 N HNO_3_ for 30 min. Furthermore, the samples were diluted with 2% HNO_3_, and macronutrients and micronutrients were analyzed using an ICP spectrometer (I) (Optima 7300DV & Avio500; Perkin-Elmer).

### Quantitative Real-Time Polymerase Chain Reaction Analysis

Pregerminating seeds of each rice genotype were grown for 3 weeks in a water cup containing soil up to the three- to four-leaf stage. These plants were treated with 150 mM (NaCl, CaCl_2_, and MgCl_2_), and RNA was extracted from their roots and shoots after 0, 6, and 12 h using a RNeasy Plant Mini Kit (Qiagen, Hilden, Germany) according to the manufacturer’s instructions. A NanoDrop 2000 spectrophotometer (Thermo Scientific, Wilmington, DE, United States) was used to measure RNA concentrations. For first-strand cDNA synthesis, the qPCRBIO cDNA Synthesis Kit and 600 ng total RNA were used. For quantitative real-time polymerase chain reaction (qRT-PCR), this study used the StepOnePlus™ Real-Time PCR System, Life Technologies Holdings Pte Ltd. (Singapore), BioFACT™ 2X Real-Time PCR Master Mix (including SYBR^®^ Green I; www.bio-ft.com; South Korea), and primers specific to the selected genes (Table 1). *OsActin1* (accession no. AB047313) was used as an internal reference gene for normalization.

**TABLE 1:** Primer list for polymerase chain reaction analysis.

Accession no.	Gene	Forward primer (5′–3′)	Reverse primer (5′–3′)	Fragment size (bp)
AB061311	*OsHKT1*	TCGGCAAGCACTGTGATAAG	CGCTTGCTCCTCTTCAAATC	98
AB021878	*OsNHX1*	ATTGGGGAATCTGTTTGCTG	ACAGACAGCTAGGCCCAGAA	84
AY785147	*OsSOS1*	GGCAGGATAATGTGGTGCTT	TGAGCAGCAGGCAATATCAC	70
AB047313	*OsActin1*	CGTCCTCCTGCTTGTTTCTC	TAGGCCGGTTGAAAACTTTG	72

### Glutathione and Peroxidase

Rice genotypes were grown for 3 weeks in a water cup containing soil up to the three- to four-leaf stage. To determine the activity of GSH and POD these plants were treated with 150 mM (NaCl, CaCl_2_, and MgCl_2_), and the leaf samples were collected after 24 h. Two hundred milligrams (FW) of frozen tissues were ground immediately in liquid nitrogen. Briefly, 200 mg samples were homogenized in 2 mL of 10% trichloroacetic acid (TCA), followed by centrifugation at 10,000 × g for 15 min. The 0.1 mL supernatant was transferred to 3 mL of 150 mM monosodium phosphate buffer, and 0.5 mL Ellman’s reagent, followed by incubation at 30*^o^*C for 5 min. Afterward, the absorbance was measured spectrophotometrically at 420 nm. The POD value was measured according to the method described by Khan et al. (2019). Briefly, 500 mg plant samples were ground immediately in liquid nitrogen. Afterward, 0.1 M potassium phosphate buffer with (6.8 pH) was added to the samples and centrifuged at 4*^o^*C for 15 min at 5,000 r.p.m. A reaction mixture contained 0.1 M potassium phosphate buffer (pH 6.8), 50 μl pyrogallol (50 μM), and 50 μl H_2_O_2_ (50 μM), were mixed with 100 μl of the sample crude extract, and the reaction mixture was incubated at 25°C for 5 min. After incubation 5% H_2_SO_4_ (v/v) was added to stop the enzymatic reaction. The resulting absorbance was measured spectrophotometrically at 420 nm. One unit of POD was directly measured by an increase of 0.1 units of absorbance.

### Statistical Analyses

Statistical analysis was performed for three replicates, where each replication was considered a block and arranged in different Petri dishes or pots in the control growth room conditions. The experiment was repeated three times. Differences among treatment means were evaluated using Duncan’s multiple range test with the significance set at *p* < 0.05. Data analysis was conducted in SPSS (IBM SPSS Statistics version 22). Figures were produced using GraphPad Prism version 9.0 (Graph Pad Software, Inc., San Diego, CA, United States).

## Results

### Effects of Different Salts on Seed Germination and Seedling Growth

**FIGURE 1:**
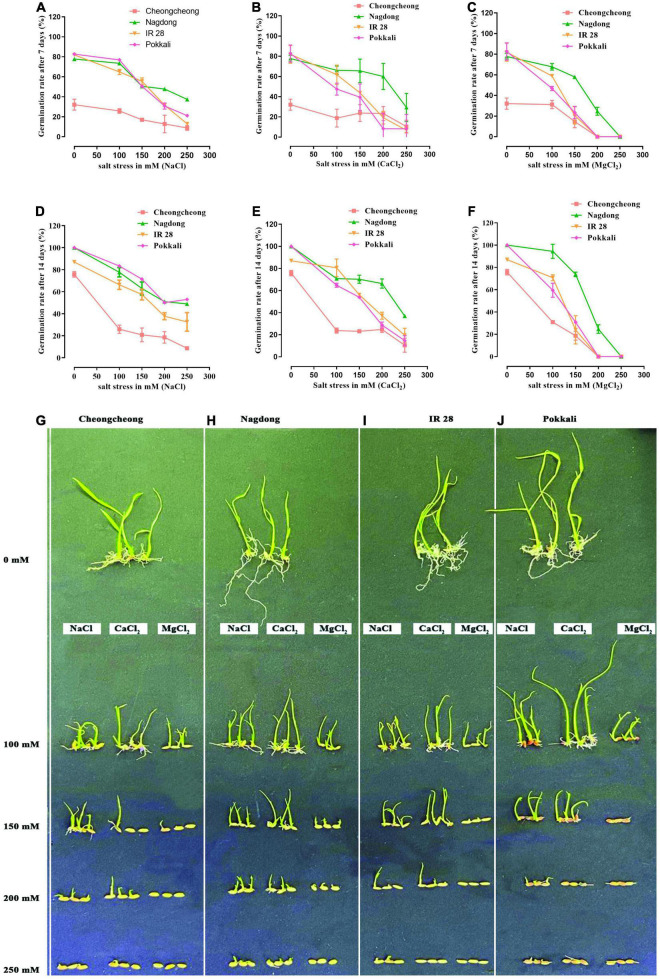
Seed germination test among rice genotypes under different salt stress. Different concentrations of different salts (NaCl, CaCl_2_, and MgCl_2_) were used to evaluate the seed germination and seedling growth **(A–J)**.

The germination rate of four rice genotypes was observed after 7 and 14 days under three different salt stresses (NaCl, CaCl_2_, and MgCl_2_) at concentrations of 100, 150, 200, and 250 mM (Figure 1). After 7 days of different salt stresses, a uniform nature of seeds germination was observed for cultivar Pokkali and IR 28 up to 150- and 200-mM, whereas Nagdong showed a high germination rate compared to other cultivars (Figures 1A–C). In contrast, the Cheongcheong germination rate was lower than other cultivars. Under CaCl_2_ stress, their germination was slightly higher than that of NaCl and MgCl_2_ stresses (Figure 1B). Finally, after 14 days, up to 250 mM NaCl and CaCl_2_ stress seeds of Pokkali showed >60 and >20% germination rates, respectively. However, Pokkali seeds can germinate only up to 150 mM MgCl_2_ stress (Figures 1D–F). IR 28 seeds up to 250 mM germinated 40% in CaCl_2_ (Figure 1E) stress and <40% in NaCl stress; similarly, up to 150 mM, it can germinate to <40% in MgCl_2_ (Figures 1D,F). Under different salt stress, the highest germination rate was recorded in Nagdong compared to other cultivars and lowest in Cheongcheong. Nagdong seeds germinated 60 and 40% up to 250 mM NaCl and CaCl_2_ stress, respectively, whereas, >30% of Nagdong seeds germinated up to 200 mM MgCl_2_ stress (Figures 1D–F). Maximum seedling growth up to 200 mM under different salt stress was observed in Nagdong compared to other cultivars. MgCl_2_ stress from 150 to 250 mM significantly inhibited seed germination and seedling growth in all cultivars, except Nagdong (Figure 1H). Similarly, under 200 mM CaCl_2_, Cheongcheong, and salt-sensitive IR 28 seedlings responded well-compared to NaCl and MgCl_2_ (Figures 1C,G,I). In contrast, among different salt stress, Pokkali seedlings responded well to NaCl only (Figure 1J).

### Growth Determination in Soil Under Different Salt Stress

**FIGURE 2:**
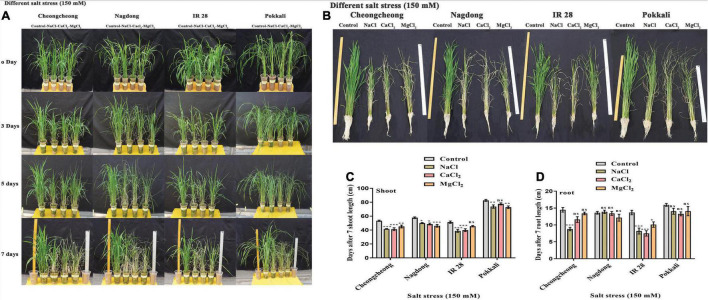
Phenotypic evaluation of rice genotypes under 150 mM of different salts (NaCl, CaCl_2_, and MgCl_2_) in a greenhouse. **(A)** Effects of different salts after 0, 3, 5, and 7 days. **(B)** Measurement of the growth index of each rice cultivar. **(C,D)** Shoot and root length determination. Statistical analysis was calculated by the Bonferroni posttest two-way repeated-measures analysis of variance. Asterisks in the vertical bar indicate a significant difference at *p < 0.05, **p < 0.01, ***p < 0.00, compared to control.

Seedling growth of four rice cultivars was determined after 7 days of 150-mM continuous stress of different salts (NaCl, CaCl_2_, and MgCl_2_). A phenotypical difference was observed among rice genotypes after 0, 3, 5, and 7 days (Figure 2A). After 3 days of stress, the most growth reduction in Cheongcheong and IR 28 was examined. After 5 days of continuous salt stress, the leaf apex and base of the tillers started burning and became dry. Finally, at 7 days, the growth rate was drastically reduced in all rice genotypes compared to control. However, Cheongcheong and IR 28 responded well to MgCl_2_ stress in soil compared to NaCl and CaCl_2_ stress. In contrast, Nagdong and Pokkali seedlings showed more vigor under NaCl and CaCl_2_ stress than MgCl_2_ (Figure 2A). Similarly, the root and shoot length was measured after 7 days of salt stress. Shoot length significantly decreased in all rice genotypes compared to their respective control. However, under different salt stress, Cheoncheong and IR 28 shoots were reduced in the same way as to control plants. The shoot length in MgCl_2_ was higher in Cheoncheong and IR 28 than NaCl and CaCl_2_ stress (Figure 2C). In contrast, Nagdong and Pokkali shoot length significantly decreased under MgCl_2_ stress compared to NaCl and CaCl_2_ stress. However, the shoot length of Nagdong and Pokkali was higher than that of Cheongcheong and IR 28 (Figure 2C). Under different salt stress, the root length of IR 28 significantly decreased compared to their respective control (Figure 2D). Similarly, NaCl stress significantly decreased the root length in cultivar Cheongcheong, whereas, the root length of Cheongcheong was non-significant on CaCl_2_ and MgCl_2_ stress compared to control plants (Figure 2D). In contrast, under different salt stress, there was no significant difference in the root length of Nagdong and Pokkali compared to control plants (Figure 2D).

### Effects of Salt Stress on the Fresh and Dry Weight of Rice

**FIGURE 3:**
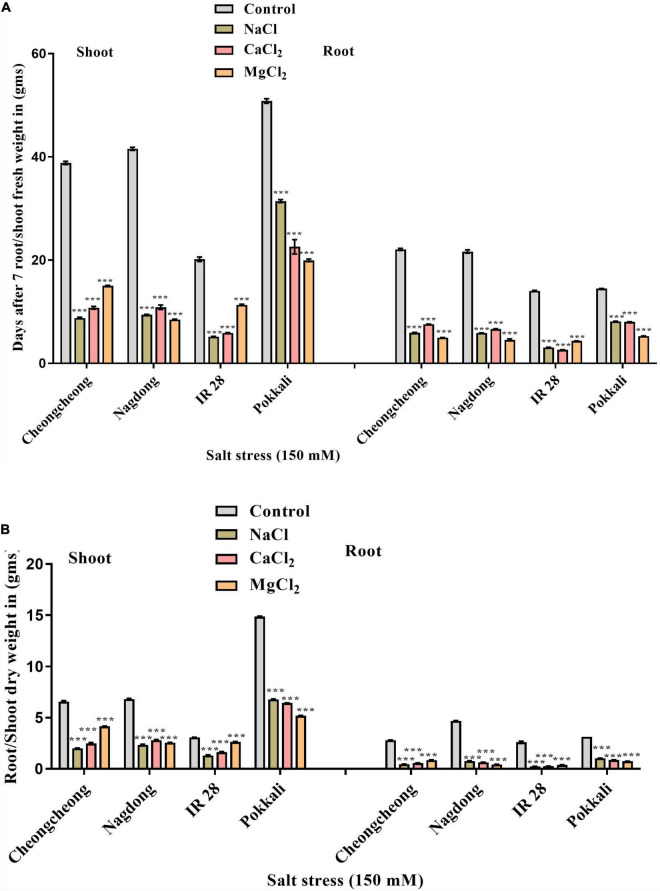
Effect of 150 mM of different salts on the shoot and root fresh and dry weight. **(A)** Fresh weight of shoots and roots. **(B)** Shoot and root dry weight. Statistical analysis was calculated by the Bonferroni posttest two-way repeated-measures analysis of variance. Asterisks in the vertical bar indicate a significant difference at ***p < 0.00, compared to control.

In soil, salinity stress significantly reduced the fresh and dry weight of the tested genotypes compared to control plants. However, Pokkali and Nagdong exhibited the highest root/shoot fresh and dry weight when exposed to different salt stress compared to the rest of the cultivars. Nagdong and Pokkali responded well under NaCl and CaCl_2_, whereas, MgCl_2_ stress significantly reduced the root/shoot fresh and dry weight (Figures 3A,B). However, under MgCl_2_ stress, Cheongcheong and IR 28 responded well and showed the highest root/shoot fresh and dry weight compared to NaCl and CaCl_2_ stress (Figures 3A,B).

### Effect of Different Salts on Nutrients Uptake

Under different salt stress, eight elements (namely K^+^, P, Ca^2+^, Mg^2+^, Na^+^, Zn^2+^, Mn^2+^, and Fe^2+^) were determined in the roots and shoots of control and treated rice plants (Tables 2, 3). Different salts stress differentially regulated the nutrients and their uptake in all rice genotypes. After 7 days of 150 mM of different salt stress, there was a high uptake of K^+^ and P ions was observed in the roots of Nagdong and Cheongcheong; however, P uptake in the roots of Cheongcheong under MgCl_2_ stress significantly decreased by 45.9-fold compared to control plants. The K^+^ and P uptake levels in the shoots of Cheongcheong, IR 28, and Nagdong significantly decreased when subjected to different salt stress. Under different salts stress, roots of IR 28 show significantly a high uptake of K^+^, but P uptake decreased significantly under CaCl_2_ and MgCl_2_ salts. However, significantly a high uptake of P was observed in the roots of Pokkali under three different salt stress compared to control plants. Similarly, under CaCl_2_ and MgCl_2_ stress, a maximum uptake of K^+^ and P was observed in the roots of Pokkali. Although CaCl_2_ and MgCl_2_ stress decreased significantly the uptake of K^+^ and P in the shoot of Pokkali, whereas in the shoot region NaCl stress significantly increased the uptake of K^+^ and P. Interestingly among all rice cultivars roots and shoots we observed that both CaCl_2_ and MgCl_2_ stress, significantly increased their respective ions compared to control plants e.g., CaCl_2_ salt increase Ca^2+^ ion and MgCl_2_ increase Mg^2+^ ion. However, different salt stresses significantly increase the uptake of Ca^2+^ and Mg^2+^ ions in the shoot of Cheongcheong, IR 28, and Nagdong except for the cultivar Pokkali (Tables 2, 3).

**TABLE 2:** Effects of different salt stress on nutrients uptake in soil among rice cultivar roots.

Cultivars	Element	Concentration (mg/kg)				*p*-value
		Control	Nacl	CaCl_2_	MgCl_2_	
Cheongcheong	K^+^	11101.3 ± 970.2^c^	16628.0 ± 72.9^b^	24169.9 ± 153.8^a^	11429.7 ± 580.4^c^	<0.001**
	P	2171.3 ± 93.6c	3338.9 ± 76.3^a^	2394.5 ± 48.6^b^	1502.0 ± 45.9^d^	<0.001**
	Ca^2+^	6703.3 ± 619.5^b^	1934.7 ± 77.7^c^	23140.2 ± 65^a^	2003.6 ± 56.5^c^	<0.001**
	Mg^2+^	5127.9 ± 272.6^b^	4017.6 ± 63^c^	3160.6 ± 37.2^d^	9615.4 ± 408.5^a^	<0.001**
	Na^+^	4080.1 ± 492.9^b^	28686.5 ± 8.9^a^	4443.5 ± 54.1^b^	2810.0 ± 143.7^c^	<0.001**
	Zn^2+^	66.1 ± 2.6^b^	83.3 ± 1.3^a^	77.0 ± 3.1^a^	52.6 ± 1.6^c^	0.001**
	Mn^2+^	245.8 ± 15.1^b^	220.7 ± 8.9^b^	109.9 ± 3.0^c^	313.5 ± 41.7^a^	0.004**
	Fe^2+^	6671.7 ± 114.1^b^	6950.6 ± 68.4^b^	4083.9 ± 255.3^c^	9499.2 ± 642.2^a^	0.001**
IR28	K^+^	11612.8 ± 413.6^bc^	10573.7 ± 472.8^c^	16134.9 ± 234.5^a^	12972 ± 852.9^b^	0.002**
	P	2259.9 ± 17.9^a^	2262.3 ± 89.8^a^	2163.9 ± 26.6^a^	1955.6 ± 28.3^b^	0.01**
	Ca^2+^	3118.4 ± 75.8^b^	2024 ± 80.5^c^	21948.7 ± 70.5^a^	1959.4 ± 64.6^c^	<0.001**
	Mg^2+^	4037.9 ± 75^b^	2751.9 ± 114.4^b^	3281.1 ± 2.4^b^	16560 ± 1060.4^a^	<0.001**
	Na^+^	4709.0 ± 233.1^b^	29443.7 ± 1222^a^	5946.8 ± 79.5^b^	4281.4 ± 289.7^b^	<0.001**
	Zn^2+^	84.1 ± 0.7^a^	64.0 ± 1.9^b^	87.8 ± 0.2^a^	52.7 ± 2.0^c^	<0.001**
	Mn^2+^	255.5 ± 1.8^a^	193.9 ± 9.6^b^	185.7 ± 1.6^b^	114.2 ± 0.8^c^	<0.001**
	Fe^2+^	6075.2 ± 93.7^a^	3817.8 ± 156.3^b^	2827.6 ± 24.3^c^	4174.4 ± 206.8^b^	<0.001**
Nagdong	K^+^	10159.7 ± 203.2^d^	12017.5 ± 30.7^c^	19113.3 ± 172.3^a^	14611.2 ± 244.3^b^	<0.001**
	P	1368.2 ± 10.7^c^	2955.4 ± 177.9^b^	2754.6 ± 25.3^b^	4036.5 ± 87^a^	<0.001**
	Ca^2+^	5342.7 ± 21.5^b^	2231.5 ± 206.9^c^	21925.9 ± 469.8^a^	1751.2 ± 17.6^c^	<0.001**
	Mg^2+^	6479.5 ± 82.3^b^	3825.2 ± 279.1^c^	4641.8 ± 208.7_*c*_	17599.3 ± 720.8^a^	<0.001**
	Na^+^	4279.7 ± 54.7^b^	18570 ± 912.1^a^	4494.2 ± 73.3^b^	3399.2 ± 50.9^b^	<0.001**
	Zn^2+^	62.3 ± 2.6^c^	98.8 ± 4.7^a^	61.1 ± 0.5^c^	71.4 ± 0.6^b^	<0.001**
	Mn^2+^	315.7 ± 23^a^	290.1 ± 57.4a^b^	224.3 ± 11.1^b^	132.1 ± 2.6^c^	0.015**
	Fe^2+^	19482.3 ± 3156.8^a^	8230.4 ± 688.1^b^	9078.2 ± 19.7^b^	3398.7 ± 69.1^c^	0.002**
Pokkali	K^+^	11056.3 ± 176.1^c^	10496.7 ± 197.7^c^	16014.5 ± 711.1^a^	12287.2 ± 203.2^b^	0.001**
	P	1403.0 ± 38.9^d^	1868.8 ± 87.2^c^	2680.7 ± 82.7^b^	2920.9 ± 48.5^a^	<0.001**
	Ca^2+^	3461.6 ± 138.1^b^	1897.5 ± 17.6^c^	19178.9 ± 686.5^a^	1298.0 ± 14.4^c^	<0.001**
	Mg^2+^	4200.5 ± 183.3^b^	4090.2 ± 91.8^b^	3154.3 ± 238.5^c^	16291.3 ± 242.9^a^	<0.001**
	Na^+^	4606.1 ± 157.9^c^	23562.1 ± 382.6^a^	7203.4 ± 272.8^b^	4215.7 ± 77.0^c^	<0.001**
	Zn^2+^	46.3 ± 3.9^b^	43.8 ± 1.3^b^	47.8 ± 1.2^b^	62.5 ± 0.5^a^	0.003**
	Mn^2+^	294.1 ± 14.9^a^	220.1 ± 23.7^b^	224.6 ± 9.8^b^	221 ± 6.9^b^	0.02**
	Fe^2+^	4901.8 ± 956.3^b^	8296.8 ± 576.9^a^	4428.5 ± 351.8^b^	6079.6 ± 221.9^b^	0.01**

Data are the mean ± standard deviation. Different letters after data (a–d) indicate significant differences among salt treatments. Means with the same letters are not significantly different by Duncan’s multiple range test at p < 0.05. **Significant at the 0.01 level.

**TABLE 3:** Effects of different salt stress on nutrients uptake in soil among rice cultivar shoots.

Cultivars	Element	Concentration (mg/kg)				*p*-value
		Control	Nacl	CaCl_2_	MgCl_2_	
Cheongcheong	K^+^	51963.5 ± 2289.0^a^	26994.1 ± 68.0^d^	32351.2 ± 2936.2^c^	38694.2 ± 175.2^b^	<0.001**
	P	5635.7 ± 146.8^a^	4093.7 ± 130.1^b^	4371.7 ± 48.5^b^	4387.7 ± 52.2^b^	<0.001**
	Ca^2+^	3009.5 ± 118.1^b^	4343.3 ± 62.2^b^	18709.2 ± 1338.1^a^	3171.7 ± 118.1^b^	<0.001**
	Mg^2+^	2754.2 ± 60.3^bc^	3077.6 ± 54.8^b^	2572.8 ± 209.1^c^	14788.2 ± 121.7^a^	<0.001**
	Na^+^	1286.5 ± 50.6^d^	26419.5 ± 118.7^a^	1900.0 ± 167.3^c^	2735.1 ± 36.6^b^	<0.001**
	Zn^2+^	51.1 ± 1.0^b^	65.4 ± 0.4a^b^	56.0 ± 1.0^b^	93.3 ± 21.5^a^	0.001**
	Mn^2+^	702.9 ± 43.0^a^	576.1 ± 19.6^b^	576.2 ± 1.8^b^	532.1 ± 1.5^b^	0.004**
	Fe^2+^	148 ± 0.3^b^	140.6 ± 2.9^b^	113.9 ± 5.8^c^	375 ± 17.2^a^	0.001**
IR28	K^+^	44429.7 ± 1363.4^a^	26970.7 ± 157.7^d^	31094.6 ± 174.1^c^	34571.9 ± 152.8^b^	0.002**
	P	6019.6 ± 65.1^a^	3390.9 ± 68.4^b^	3268.9 ± 100.7^b^	2907.4 ± 23.9^c^	0.01**
	Ca^2+^	3676.9 ± 129.7^c^	5287.4 ± 40.1^b^	28988.2 ± 959.9^a^	5087.4 ± 62.2^b^	<0.001**
	Mg^2+^	2458.5 ± 80.3^d^	3165.8 ± 2.4^c^	3346.1 ± 87.5^b^	11731.2 ± 41.4^a^	<0.001**
	Na^+^	725.1 ± 23.6^c^	27101.0 ± 14.1^a^	2057.3 ± 10.7^b^	550.6±9^d^	<0.001**
	Zn^2+^	49.9 ± 1.1^a^	50.5 ± 1.0^a^	50.0 ± 1.4^a^	43.4 ± 0^b^	<0.001**
	Mn^2+^	547.6 ± 8.6^b^	511.6 ± 1.4^c^	557.3 ± 6.1^ab^	564.9 ± 3.4^a^	<0.001**
	Fe^2+^	153.3 ± 35.4^a^	107.2 ± 1.1^a^	121.4 ± 4.4^a^	107.2 ± 13.8^a^	<0.001**
Nagdong	K^+^	36522.2 ± 174.8^a^	35014.8 ± 1976.9^ab^	31972.7 ± 1881.7^b^	31370 ± 1411.7^b^	<0.001**
	P	5643.5 ± 51.6^a^	4443.2 ± 63.2^c^	4642.2 ± 60.8^b^	3831.9 ± 21.3^d^	<0.001**
	Ca^2+^	2731.6 ± 36.4^b^	3417.8 ± 107.1^b^	27298.8 ± 1539.0^a^	3686.7 ± 44.3^b^	<0.001**
	Mg^2+^	3044.0 ± 29.0^b^	3170.9 ± 103.6^b^	3374.8 ± 169.6^b^	15613.6 ± 240^a^	<0.001**
	Na^+^	550.7 ± 3.3^b^	32379.1 ± 2017.6^a^	1552.9 ± 105.6^b^	1470.3 ± 53^b^	<0.001**
	Zn^2+^	38.8 ± 0.1^d^	60.5 ± 0.1^a^	44.9 ± 0.3^c^	56.1 ± 1.1^b^	<0.001**
	Mn^2+^	503.7 ± 7.2^c^	610.9 ± 12.4^b^	654.5 ± 14.1^a^	615.6 ± 5.8^b^	0.015**
	Fe^2+^	162.4 ± 7.5^a^	126.1 ± 8.9^a^	123.0 ± 39.3^a^	122.6 ± 10.4^a^	0.002**
Pokkali	K^+^	45199.6 ± 1099.5^b^	78613.3 ± 1934.2^a^	40543.6 ± 437.3^c^	40703.8 ± 521.7^c^	0.001**
	P	4396.0 ± 11.5^b^	5015.3 ± 54.6^a^	3268.6 ± 37.7^d^	3722.2 ± 32.9^c^	<0.001**
	Ca^2+^	3458.3 ± 48.0^b^	2050.3 ± 55.0^d^	12541.5 ± 31^a^	2926.1 ± 12^c^	<0.001**
	Mg^2+^	3455.6 ± 50.0^c^	3996.6 ± 111.9^b^	2896.4 ± 9.6^d^	8642.9 ± 131.9^a^	<0.001**
	Na^+^	583.5 ± 19.6^b^	11561.7 ± 366.5^a^	810.3 ± 2.7^b^	947.4 ± 4.9^b^	<0.001**
	Zn^2+^	44.0 ± 1.1^c^	58.2 ± 2.2^a^	42.5 ± 0.5^c^	51.6 ± 1.1^b^	0.003**
	Mn^2+^	1145.2 ± 13.5^c^	1241.3 ± 11.7^b^	1283.7 ± 0.7^a^	1277.7 ± 5.3^a^	0.02**
	Fe^2+^	159.3 ± 6.7^b^	464.3 ± 88.7^a^	77.3 ± 1.5^b^	99.3 ± 7.7^b^	0.01**

Data are the mean ± standard deviation. Different letters after data (a–d) indicate significant differences among salt treatments. Means with the same letters are not significantly different by Duncan’s multiple range test at p < 0.05. **Significant at the 0.01 level.

Tables 2, 3 show that the micronutrient Na^+^ significantly increased by NaCl stress in the roots and shoots of all rice genotypes compared to control plants under different salt stress. Both NaCl and CaCl_2_ salts significantly increase the Zn^2+^ uptake in the roots of Cheongcheong and shoot of IR 28. However, among all rice cultivars under NaCl stress, a remarkable uptake of Zn^2+^ was observed in the roots of Nagdong (Table 2). Under different salt stress, a maximum uptake of Mn^2+^ was observed in the shoots of Nagdong, Pokkali, and IR 28, whereas in the root of Cheongcheong MgCl_2_ significantly increased the uptake of Mn^2+^. However, under different salt stress, Mn^2+^ uptake significantly decreases in the roots of all rice cultivars. Different salt stress significantly decreased the uptake of total Fe^2+^ content in the roots and shoots of all rice genotypes, but Fe^2+^ content significantly increased under MgCl_2_ and NaCl stress in the roots and shoots of Cheongcheong and Pokkali (Tables 2, 3).

### Ion Transport-Related Gene Expression Under Different Salt Stress Among Rice Cultivars

**FIGURE 4:**
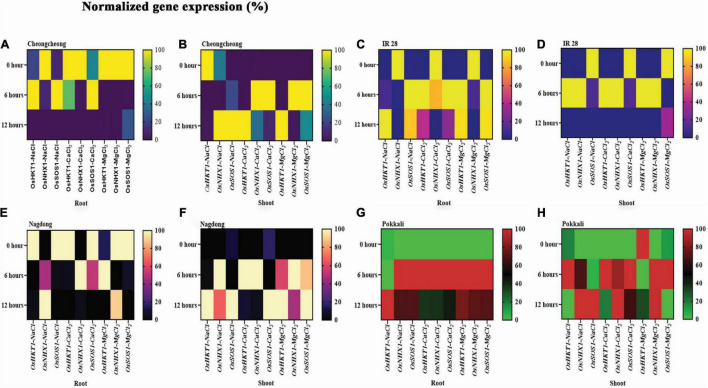
Under 150 mM of different salt stress, quantitative real-time polymerase chain reaction expression analysis of *OsHKT1*, *OsNHX1*, and *OsSOS1* relative to salinity in the roots and shoots of rice genotypes. The expression pattern in the heatmap **(A–H)** was determined in normalized gene expression (%) after 0, 6, and 12 h of salt stress.

Under different salt stress at different time points, the expression of three very important ion transport-related genes, namely *OsHKT1*, *OsNHX1*, and *OsSOS1*, was examined (Figure 4). Gene regulation under different salt stress differed in roots and shoots among rice genotypes according to StepOnePlus™ Real-Time PCR System. After 6 h of different salt stress, NaCl and CaCl_2_ upregulated *OsHKT1* and *OsSOS1* in the roots of Cheongcheong, whereas these genes were downregulated after 12 h; *OsSOS1* was 40% upregulated by MgCl_2_ in the roots of Cheongcheong (Figure 4A). In contrast, after 6 h of different salt stress, *OsNHX1* and *OsSOS1* were upregulated by CaCl_2_ and MgCl_2_ in the shoots of Cheongcheong. In addition, after 12 h of salt stress, CaCl_2_ and MgCl_2_ highly expressed *OsHKT1*, whereas *OsNHX1* and *OsSOS1* were upregulated by NaCl stress (Figure 4B). Similarly, IR 28 roots exhibited absolute expression of genes *OsHKT1* and *OsSOS1* after 6 h exposure to 150 mM salt concentrations of NaCl, CaCl_2_, and MgCl_2_, whereas after 12 h of stress, these genes were almost 90% upregulated by NaCl and MgCl_2_ (Figure 4C). Similarly, after 6 h of stress, NaCl and MgCl_2_ upregulated *OsHKT1* and *OsNHX1* in the shoots; however, these genes were significantly downregulated after 12 h of stress, except for *OsSOS1*, which was almost 40% upregulated by MgCl_2_ (Figure 4D). After 6 and 12 h of stress, NaCl and CaCl_2_ showed the most *OsNHX1* and *OsHKT1* upregulation in the roots of Nagdong, although MgCl_2_ up to 80% also induced *OsNHX1*. In contrast, NaCl, CaCl_2_, and MgCl_2_ downregulated *OsSOS1* in the roots of Nagdong (Figure 4E). After 6 h of stress, NaCl, CalCl_2_, and MgCl_2_ upregulated *OsHKT1* and *OsNHX1* in Nagdong shoots; however, after 12 h of stress, NaCl, CaCl_2_, and MgCl_2_ upregulated *OsHKT1* and *OsSOS1* (Figure 4F). Pokkali roots after 6 h of stress showed very unique results; all ion transport-related genes, such as *OsHKT1*, *OsNHX1*, and *OsSOS1*, were 100% upregulated by NaCl, CaCl_2_, and MgCl_2_, except for *OsHKT1*, which was downregulated by NaCl (Figure 4G). In addition, after 12 h of stress, the maximum *OsHKT1* upregulation was under NaCl and MgCl_2_ stress in the roots. Similarly, in the shoots of Pokkali after 6 h of stress, NaCl, CaCl_2_, and MgCl_2_ upregulated *OsHKT1* and *OsSOS1*, whereas NaCl and CaCl_2_ 100% upregulated *OsNHX1* and *OsSOS1* after 12 h of stress (Figure 4H). In contrast, after 6 and 12 h of stress, MgCl_2_ upregulated *OsNHX1* in the shoots of Pokkali (Figure 4H).

### Glutathione and Peroxidase Activity

**FIGURE 5:**
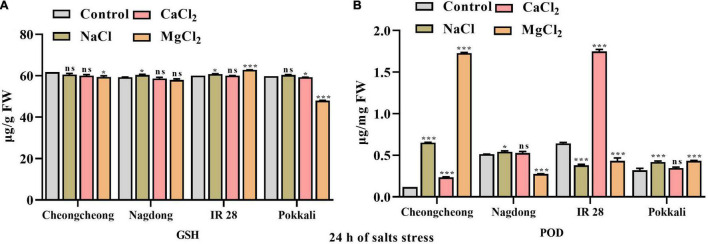
Effect of 150 mM of different salts on GSH and POD contents in the leaves of different rice genotypes. The GSH **(A)** and POD **(B)** activity was determined after 24 h of salt stress. Statistical analysis was calculated by the Bonferroni posttest one-way repeated-measures analysis of variance. Asterisks in the vertical bar indicate a significant difference at *p < 0.05, ***p < 0.00, compared to control.

After 24 h of different sat stress, GSH content was increased significantly in the leaves of IR 28 with 150 mM NaCl and MgCl_2_, and the highest GSH content was observed at MgCl_2_ salt compared to their respective control. Similarly, at 150 mM NaCl stress a higher GSH content was observed in the leaves of Nagdong. However, both CaCl_2_ and MgCl_2_ salts significantly decrease the GSH content in cultivar Pokkali whereas, in cultivar Cheongcheong CaCl_2_ significantly decreases the GSH content compared to the control group (Figure 5A). The second enzyme responsible for the deactivation of H_2_O_2_ is a peroxidase. POD activity increased significantly in both cultivar Cheongcheong and IR 28 at 150 mM MgCl_2_ and CaCl_2_ compared to Nagdong and Pokkali. However, under NaCl and CaCl_2_ salts the POD activity was found significantly higher in cultivar Cheongcheong compared to their respective control. In contrast, POD activity in IR 28 decreased significantly by NaCl and MgCl_2_ salts compared to the control. In the case of Cultivar Nagdong and Pokkali POD activity increased significantly under NaCl and MgCl_2_ salts however, MgCl_2_ salt decreased POD activity in cultivar Nagdong compared to the control group (Figure 5B).

## Discussion

The response and adaptation of rice to salt stress is a very complex mechanism. Salt stress causes leaf rolling, root growth inhibition, and reduced plant height, tiller number, and spikelet sterility, leading to yield reduction (Ali et al., 2014; Razzaq et al., 2020). A previous study reported that salinity stress up to 20 ds m^–1^ dynamically inhibited rice seed germination, root and shoot length growth reduction, dry matter, and ultimately yield reduction (Hakim et al., 2010). However, another study demonstrated that rice seeds have a salt-tolerant ability up to some extent at germination and, in some cases, are not seriously affected by salts up to 16.3 ds m^–1^ (Khan et al., 1997). A previous study suggested that NaCl and its mixtures (MgSO_4_ + NaCl and Na_2_SO_4_ + CaCl_2_) significantly inhibited seed germination and root length in sugar beet cultivars with EC up to 8 and 4 ds/m, respectively (Jafarzadeh and Aliasgharzad, 2007). This study showed a higher reduction in seed germination and shoot and root length of rice cultivars by NaCl, along with CaCl_2_ and MgCl_2_, similar to findings for NaCl and Na_2_SO_4_ (Stewart, 1898). Bonilla et al. (2004) demonstrated high Ca content in moderate salinity levels up to 75 mM NaCl increased the proportion of germinated pea seeds and recovered the delay in germination. This study found a high germination percentage on Cheongcheong and IR 28; thus, this might be the role of Ca in CaCl_2_ (Figure 1E). A previous study reported that the germination percentage of sugar beet and cotton is significantly affected by NaCl, CaCl_2_, and Na_2_SO_4_, with a high extent or equal to 200 mM (Shonjani, 2002). In this study, under different salt stress, Cheongcheong and IR 28 significantly inhibited seed germination to ≥200 mm, contrary to Nagdong and Pokkali (Figures 1D–F). In current work under different salts stress cultivar Nagdong show significantly higher germination percentage and good seedlings growth compared to other cultivars. However, salt-sensitive cultivar IR 28, and Cheongcheong show high seeds germination under CaCl_2_ salt compared to the other two salts NaCl, and MgCl_2_. Thus, based on current work we believe that cultivar Nagdong has more salt-tolerant ability, and it should be used as a salt-tolerant cultivar for future studies.

Salinity stress reduced rice plant height, roots, and shoot dry matter at various growth phases (Puvanitha and Mahendran, 2017). NaCl treatment most prominently decreased the shoot growth in rice, sugar beet, and cotton compared to root growth (Shonjani, 2002). Another study demonstrated that salinity stress drastically reduced plant height, root and shoot dry weight, and yield of rice cultivars (Puvanitha and Mahendran, 2017). Many studies showed that the fresh and dry weights of the shoot and root system are affected negatively by changes in salinity concentration, depends on type of plant species, or type of salt present (Taffouo et al., 2010). In this study, different salt stress significantly affected rice plant height, root, and shoot dry matter compared to control. It might be due to the osmotic effect because in saline soil roots exclude all Na^+^ and Cl^–^ ions while taking up water; the high concentration of these ions may lead to the reduction or death of the plant (Figures 2A–D). These findings agreed with Jamil et al. (2007). Growth reduction may be due to the toxic effects of NaCl or unbalanced nutrient uptake by the seedlings (Datta et al., 2009).

During salinity stress, a high concentration of Na^+^ and a higher Na^+^/K^+^ ratio disrupt ion homeostasis and decrease other nutrients (Simaei et al., 2012; Bose et al., 2014; Rahman et al., 2016b). Salt stress more seriously affects the ion balance of Na^+^, K^+^, and Cl^–^ in old rice leaves compared to young leaves (Wang et al., 2012). In this study, the high accumulation of Na^+^/K^+^ ions among rice cultivars altered other macronutrients and micronutrients. Some studies reported that, as salinity level increases, Mn content increased in sunflower shoots compared to roots (Achakzai et al., 2010). The availability of most nutrients depends on the pH of the soil as well the nature of binding sites on organic and inorganic particle surfaces. In saline soils, the solubility of micronutrients such as Fe^2+^, Mn^2+^, Zn^2+^, and Cl^–^ is particularly low, and plants grown in these soils often experience a deficiency in these elements (Pessarakli, 2019). Although the micronutrient concentration in plant shoots may increase, decrease, or have no effect depending on salinity, the salt tolerance of plant species, type of plant tissue, micronutrient concentration, environmental conditions, or sudden changes in the permeability of the plant cell membranes. Previous studies reported that salt-stress conditions decreased Mn content due to disrupted ion imbalance (Tunçturk et al., 2008; Wu and Wang, 2012). However our results indicated a high Mn content in the shoots of Nagdong and Pokkali, whereas varietal response was inconsistent; therefore, under different salt stress, cultivar Cheongcheong and IR 28 showed low Mn content in roots and shoots (Tables 2, 3). Thus it might be possible that the salt-tolerant ability of both cultivars Nagdong and Pokkali links with high Mn content instead of cultivar Cheongcheong and IR 28. Another study reported that exogenous application of Mn induced salt stress tolerance in rice seedlings (Rahman et al., 2016a). In present study under different salt stress, both cultivar Nagdong and Pokkali show high Mn content that might be induced salt stress tolerance in these cultivars compared to cultivars Cheongcheong and IR 28. A previous study reported that a high Na^+^ concentration has a contrasting effect on K^+^ ion, an essential plant nutrient for plant growth and development (Jung et al., 2009).

High Na^+^ in plant cells seriously damages membrane systems and organelles, resulting in abnormal plant growth and development and plant death (Quintero et al., 2007; Siringam et al., 2011). This study revealed the inconsistent uptake of Na^+^/K^+^ in the roots among rice cultivars under different salt stress but consistent increase and decrease between Na^+^/K^+^ ions in the shoots; thus, this unbalanced regulation of ions might be the cause of lower plant growth and fresh and dry matter. These findings agreed with Eker et al. (2006). Another study also reported the high uptake of Na^+^ over K^+^ ions in plant tissue of *Butea monosperma* (Hirpara et al., 2005). A previous study suggested that in a higher Na^+^/K^+^ ratio the K^+^ efflux activates non-selective cation channels (NSCCs) and guard cell outward rectifying potassium (GORK) channels, and K^+^ leakage also leads to a higher reactive oxygen species production that might decrease Mg^2+^, Mn^2+^, and Zn^2+^ contents (Demidchik et al., 2002). In this study, among different rice cultivars, NaCl and CaCl_2_ stress decreased Mg and Mn contents in the roots, whereas Zn and these elements differentially increased or decreased in the shoots of different genotypes (Tables 2, 3).

The phenomenon of increasing Ca^2+^ ions under mixed salt was also observed in New Zealand spinach (*Tetragonia tetragonioides*, Pall) and red orach (*Atriplex hortensis* L.) (Wilson et al., 2000). Another study demonstrate that an initial Ca^2+^ signal after salt stress was formed close to the root apex and then appeared to disperse to basal parts of this organ (Moore et al., 2002). In this study, CaCl_2_ stress mainly increased the Ca^2+^ level in the roots of different rice cultivars and the shoots of Pokkali. Several studies observed that the salt-induced Ca^2+^ rise originated within the roots (Kiegle et al., 2000; Tracy et al., 2008). However, under different salt stress, a high accumulation of Ca^2+^ ions was found in the shoots of Cheongcheong, Nagdong, and IR 28 compared to control (Table 3). Thus, these findings agreed with the above-mentioned statements that Ca^2+^ ions might be alleviate the toxic effects of Na^+^ ions because the Ca^2+^ ion concentration significantly decreased under NaCl and MgCl_2_ stress in Pokkali. A high salt level in rice decrease the Na-Ca selectivity (Zeng et al., 2003). Thus Na-Ca selectivity might be one salt tolerance component and effective selection criterion in screening for salt tolerance (Tables 2, 3). However, the exact phenomenon that Ca^2+^ ion alleviate the toxic effect of Na^+^ unknown, but recent findings put forward that the function of this second messenger in salt stress responses may be even more adaptable than so far appreciated. Indeed, accumulating evidence points to the involvement of a diverse array of Ca^2+^ sensor proteins in the various aspects of salinity tolerance. For example, CML9 [a member of the calmodulinlike (CML) gene family] was found to be up-regulated during salt stress, and *cml9* loss-of-function mutants displayed hypersensitivity in germination assays on medium containing either NaCl or ABA, whereas adult *cml9* plants show enhanced tolerance toward irrigation with salt water (Magnan et al., 2008).Thus, salinity stress either increases or decreases the uptake of micronutrients. High NaCl concentration generally increases Cu, Fe, and Zn contents (Alam, 1999; Víllora et al., 2000). Differences can be evaluated to plant species, plant tissue, environmental conditions, and salinity level. Another study reported that high salt stress decreases Ca^2+^, K^+^, Mg^2+^, P, Mn, and N contents (Talei et al., 2012). In saline soil micronutrients availability to plants is relatively low, and plants grown in such soil show nutrient deficiency in the plant body (Page, 1996). In this study, NaCl stress decreases the concentrations of all micronutrients (Fe^2+^, K^+^ Mn^2+^, Mg^2+^, P, Zn) in the roots and shoots of different rice cultivars. However, in the roots and shoots of different genotypes, these elements are differentially regulated by CaCl_2_ and MgCl_2_ (Tables 2, 3).

This study focused on *OsHKT1*, *SOS1*, and *NHX1*, three very important genes, and studied their expression pattern in the roots and shoots of rice cultivars with different time points. These genes are currently the most extensively studied mechanisms in controlling the salt stress response in plants. *Thellungiella salsuginea* comprised at least two *HKT* genes. *TsHKT1;1* is expressed at very low levels, whereas *TsHKT1;2* is transcriptionally mainly upregulated by salt stress (Ali et al., 2012). Another study demonstrated that, under NaCl stress, the *OsHKT1* transcript was significantly downregulated in salt-tolerant cultivar. Pokkali but upregulated in salt-sensitive cv. BRRI Dhan29 shoot after 6 h intervals (Kader et al., 2006). However, in this study, NaCl stress after 6 and 12 h upregulated *OsHKT1* in the roots and shoots of Pokkali and IR 28 (Figures 4C,D,G,H). Under NaCl stress, *OsHKT1* was upregulated conversely in the roots of Cheongcheong and shoots of Nagdong at 6 and 12 h intervals (Figures 4A,F). In the same way, NaCl stress downregulated *OsHKT1* in the roots of Nagdong and shoots of Cheongcheong (Figures 4B,E). We believe that under salt stress, *OsHKT1* up-regulation might be closely link with Na^+^ concentration because in root the of Cheongcheong we observed high level of Na^+^ ion than that of cultivar Nagdong root. Conversely, in the shoot of Nagdong have a high Na^+^ ion compared to cheongcheong. Thus, it might possible that high accumulation of Na^+^ leads a high induction of *OsHKT1*. A previous study reported that, under high NaCl stress, high Na^+^ leads to K^+^ deficiency that might cause the induction of *OsHKT1* (Maathuis and Amtmann, 1999). In the current study under NaCl stress, the Na^+^ concentration is relatively higher than K^+^ in the roots of different rice genotypes. However, K^+^ ions mainly higher in the shoot of different rice genotypes accept salt-sensitive cultivar IR 28 (Tables 2, 3). Thus, the high Na^+^ level in the root or shoot and high accumulation of K^+^ in the shoot might be the reason for *OsHKT1* up-regulation. In this study, NaCl stress, along with other salts, such as CaCl_2_ and MgCl_2_, regulate *OsHKT1* differentially among rice cultivars. After 6 and 12 h of stress, *OsHKT1* was mostly upregulated by CaCl_2_ and MgCl_2_ in the roots and shoots of IR 28, Pokkali, Nagdong, and Cheongcheong; however, after 6 h of stress, CaCl_2_ upregulated *OsHKT1* in the shoots of Nagdong, IR 28, and Pokkali. In the present study both CaCl_2_ and MgCl_2_ salt increase, K^+^ content compared to Na^+^ in the root and shoot of different rice genotypes. Thus, it might be the reason for *OsHKT1* upregulation in the root and shoot region of rice genotypes. The previous study showed that, with an extended stress period, the salt-tolerant cultivar Pokkali starts to downregulate *OsHKT1* expression in roots and shoots of rice (Kader et al., 2006). Our study demonstrated that NaCl stress downregulated *OsHKT1* in the roots and shoots of cv. Pokkali after 6 and 12 h but in the roots of Pokkali after 12 h of NaCl stress it induced the expression of *OsHKT1*. Cultivar Pokkali is a salt-tolerant ability thus under salt stress initially it maintain internal Na^+^ ion in the root system. Therefore, it might show *OsHKT1* downregulation in the root and shoot of Pokkali. On the other, hand prolong NaCl stress, increase the internal Na^+^ in the root of Pokkali that might cause a high induction of *OsHKT1*.

A previous study reported high expression of *SOS1* and *NHX* under NaCl stress in the leaves and roots of *Kochia scoparia* after 48 h of stress (Fahmideh and Fooladvand, 2018). This study indicated that the expression pattern of *OsNHX1* and *OsSOS1* under different salt stress is different among rice genotypes. However, after 6 and 12 h of different salt stress, *OsNHX1* and *OsSOS1* were upregulated in the roots of cv. Pokkali and shoots of cv. Cheongcheong (Figures 4B,G). Thus, these findings agreed with Fahmideh and Fooladvand (2018). In the current study, under different salt stress, we observed that Na^+^ and K^+^ ions regulated inversely in the root and shoot of rice genotypes. The higher the Na^+^ level lower will be K^+^, and vice versa. Thus, in the root of Pokkali and shoot of Cheongcheong K^+^ ion higher over Na^+^ ion. Therefore, it might be the possible reason for *OsNHX1* and *OsSOS1* up-regulation. A previous study suggested that overexpression of *AtNHX1* enhanced the salt tolerance ability of *A. thaliana* (Apse et al., 1999). In this study, there was a high expression of *OsNHX1* in the roots and shoots of cv. Nagdong and Pokkali under NaCl stress (Figures 4E,H). Thus, the salt tolerance ability of Nogdong and Pokkali might be due to *OsNHX1* upregulation. Another study reported that the Na^+^/H^+^ antiporter *NHX* is an important membrane protein that helps pump Na^+^ into the vacuoles to minimize Na^+^ toxicity and alleviate the serious effects of salt stress (Wu et al., 2004). In the SOS pathway, *SOS1*, in particular, leads to high sensitivity to NaCl in glycophytes and halophytes (Liu et al., 2000; Oh et al., 2009). Under salt stress, high gene expression of *SOS1* was noted in the SOS3 or SOS2 mutant plant (Shi et al., 2000). In this study, under different salt stress, there was a high expression of *OsSOS1* in the roots of IR 28, Pokkali, and Cheongcheong (Figures 4A,C,G). Similarly, after 6 and 12 h of salt stress, *OsSOS1* upregulation was observed in the shoots of Cheongcheong, Nagdong, and Pokkali (Figures 4B,F,H). The ion homeostasis of the Na^+^/K^+^ ratio is a key factor see (Table 3) in the shoot of above mention cultivars that have high K^+^, compared to Na^+^. Thus, the upregulation of *OsSOS1* might be linked to high K^+^ content. In the present study under different salt stress, cultivar Nagdong root shows high Na^+^ ion over K^+^, Thus, decreased K^+^ level in the root might be Consequences of down-regulate *OsSOS1* gene. Conversely, high K^+^ ion the shoot of Nagdong show high induction of *OsSOS1* gene.

Glutathione is a strong antioxidant; hence, it has a very important role in antioxidant defense. Under adverse environmental conditions, such as drought, salinity, etc., excessive amounts of ROS are formed, which leads to oxidative stress. Further, GHS helps to maintain the reduced state of constituents of the AsA-GSH pathway and thus detoxifies ROS. The DHAR enzyme uses GSH to convert oxidized ascorbic acid (DHA) to AsA (Foyer and Noctor, 2011). Another study suggested the function of GSH as an antioxidant that confers abiotic stress tolerance is well-established, but the mechanism underlying this defense response is unknown. Cross-communication of GSH with other signaling molecules helps in adaptation to abiotic stress (Ghanta and Chattopadhyay, 2011). Plants under stress conditions, strongly control the production, and elimination of ROSs by many enzymatic and non-enzymatic processes to the alleviate their damages (Jalali et al., 2020). In the experiments presented here, after 24 h of 150 mM NaCl, and MgCl_2_ salt we observed the high GSH content in the leaves of Nagdong and salt-sensitive cultivar IR 28. In the present work at 150 mM NaCl stress, cultivar Pokkali, and Cheongcheong did not affect GSH content (Figure 5A). Under NaCl stress, GSH contents in the chloroplasts of the cultivars Pokkali and Peta decrease significantly (Wang et al., 2014). However, a significant reduction was observed here under CaCl_2_ and MgCl_2_ in cultivars Pokkali, and Cheongcheong seedlings (Figure 5A). Therefore, it might be possible that a high GSH content in cultivars Nagdong under NaCl stress, and a high GSH content in IR 28 under MgCl_2_ stress help to maintain plant vigor under salinity stress (Figures 2A–C). Another study reported that POD activity during the seedling stage increased greatly in IR 28 and AT 353 grown at 4 and 6 dS/m compared to Pokkali (Safeena and Bandara, 2006). A previous study suggested that the POD activity of plants was shown to be up-regulated to reduce oxidative stress, cell membrane damage, and altered Ca^2 +^ concentrations caused by ROS generated by salinity treatment (Sergio et al., 2012). In our study, CaCl_2_, and MgCl_2_ salt show the highest increase in POD activity in cultivars IR 28 and Cheongcheong. This increase is 2-fold higher than NaCl, as well as from their respective control. Therefore, it might be the possible reason that high POD activity under CaCl_2_ and MgCl_2_ stress reduces oxidative stress and ROS accumulation in cultivar Cheongcheong and IR 28 compared to NaCl treatment. Here we suggest that a high POD value might be involved to increase shoot growth, fresh and dry weight in cultivar Cheongcheong and IR 28 (Figures 2A–C, 3A,B).

## Conclusion

The high worldwide population and industrialization lead to climate change that causes soil salinization and ultimately affects crop production, especially rice. This study tested four rice genotypes with three different salts (NaCl, CaCl_2_, and MgCl_2_). However, each cultivar responded differently to different salts, but NaCl stress most drastically reduced the agronomic traits among all rice cultivars. Cheongcheong and IR 28 had a mostly similar response to different salts. Nagdong and Pokkali had a similar response to different salts, but Pokkali showed more resistance to NaCl stress in the soil, whereas Nagdong showed more resistance to all salt. *OsHKT1*, *OsNHX1*, and *OsSOS1* were upregulated in Nagdong and Pokkali. Under different sat stress a high induction of GSH was observed in cultivar Nagdong, and IR 28, whereas, higher POD activity was recorded in cultivar Cheoncheong and IR 28. Therefore, there is a need to develop a new overexpresser of Cheongcheong and a knockout of Nagdong in the near future using the CRISPR/Cas9 technique for plant molecular breeding laboratories in Korea.

## Data Availability Statement

The original contributions presented in this study are included in the article/supplementary material, further inquiries can be directed to the corresponding author.

## Author Contributions

MF planned, designed, and performed the research study, analyzed the data, and wrote the findings. SA helped with data collection. Y-HJ and J-RP contributed to the ICP and statistical analysis. D-DZ and E-GK contributed to the experimental resources. K-MK edited the manuscript. All authors read and approved the final manuscript.

## Conflict of Interest

The authors declare that the research was conducted in the absence of any commercial or financial relationships that could be construed as a potential conflict of interest.

## Publisher’s Note

All claims expressed in this article are solely those of the authors and do not necessarily represent those of their affiliated organizations, or those of the publisher, the editors and the reviewers. Any product that may be evaluated in this article, or claim that may be made by its manufacturer, is not guaranteed or endorsed by the publisher.
